# Acute reversible monoparesis in multiple neurocysticercosis: A case report and review of literature

**DOI:** 10.1002/ccr3.6131

**Published:** 2022-07-22

**Authors:** Bishal Dhakal, Sachin Sapkota, Sheetal Shrestha, Suchita Acharya, Aakriti Parajuli, Aashish Baniya, Raju Paudel

**Affiliations:** ^1^ Nepalese Army Institute of Health Sciences Kathmandu Nepal; ^2^ Chitwan Medical College Bharatpur Nepal

**Keywords:** focal neurological deficit, lacunar infarction, monoparesis, neurocysticercosis, space occupying lesion

## Abstract

Focal neurological deficit like monoparesis due to cortical lesions is a rare entity. In spite of the common presentations like seizures and headaches in neurocysticercosis, occurrence of reversible monoparesis is an atypical phenomenon. Even in the absence of infarct or hemorrhages, manifestation of neural deficit due to compressive effect only is an interesting finding. And on top of that, reversible nature of the deficit in space occupying lesion is a rare occurrence in the existing literature. Here, we describe a known case of neurocysticercosis with reversible acute monoparesis secondary to multiple neurocysticercosis. The variations with which neurocysticercosis can present broaden our understanding in its pathophysiology and management protocol.

## 1 INTRODUCTION

Neurocysticercosis (NCC), a form of cysticercosis, is caused by larval form of tapeworm *Taenia solium*. The common manifestations in NCC are seizures, followed by headache and some neurological deficits.^1^, ^2^ It can also lead to various neurological sequalae such as hydrocephalus and epilepsy.^1^ The variants that can be present in NCC are parenchymal and extra‐parenchymal lesions. The parenchymal lesions progress through various stages as vesicular, colloidal, granular‐nodular, and finally calcification.^1^, ^2^ The focal neurological deficit (FND) due to ischemic cerebral vascular disease has also been attributed to NCC as a cause. Among the deficits, pure motor hemiparesis (PMH) is the one ascribed to parenchymal NCC.^3^, ^4^ Lacunar infarction is often the etiology behind PMH. However, pure motor monoparesis has not been seen commonly in lacunar infarct.^5^ Apart from the cerebrovascular disease, FND like PMH and pure motor monoparesis can also be due to the space occupying lesion.^1^, ^2^, ^5^ Hence, we present a case of 42‐year‐old right‐handed male with NCC under anti‐epileptic medication presenting with reversible pure motor monoparesis.

## 2 CASE PRESENTATION

A 42‐year‐old right‐handed Hindu male, known case of seizure disorder due to neurocysticercosis (NCC), presented to the emergency department of our center in April 2022 with right upper limb weakness and slurry speech for 48 h. He denied of headache, loss of consciousness, abnormal body movements, facial deviation, trauma, fever, neck stiffness, visual symptoms, and urinary and bowel incontinence. He was under tablet valproate (500 mg twice a day) and tablet levetiracetam (500 mg twice a day) for fifteen years for seizure disorder due to NCC. There were no any risk factors for stroke etiology. He denied of hypertension, diabetes mellitus, and cardiac disease.

On general examination, he was conscious and oriented to time, place, and person. He was hemodynamically stable. The higher mental function was intact with slurry speech at the time of presentation. On motor examination, the power of right upper limb was 4/5 on medical research council (MRC) grading whilst rest of the other limbs had normal response. The biceps and supinator reflex on right upper limb had brisk response. The sensory examination was intact with decreased corneal reflex on right eye. However, occasionally he complained of tingling sensation on distal part of right upper limb. The Babinski reflex was down‐going bilaterally. There were no signs of cerebellar, meningeal, and autonomic dysfunction. However, gag reflex was absent at the time of presentation. He was admitted with the provisional diagnosis of acute monoparesis due to cerebrovascular accident for further evaluation.

**FIGURE 1:**
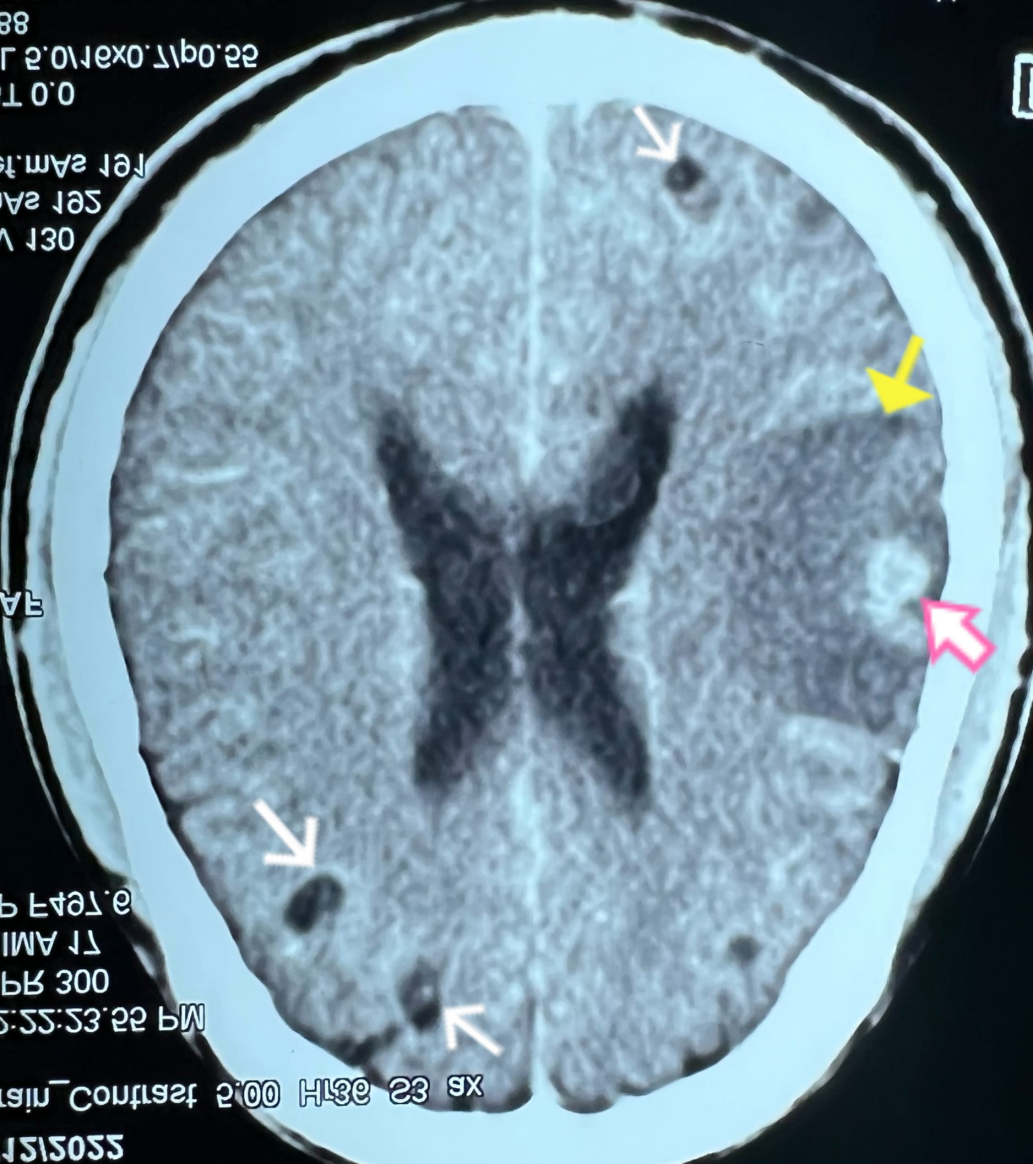
Axial CECT brain. White arrows showing multiple cystic lesions in bilateral cerebrum, Pink arrow showing calcifications, Yellow arrow showing marked perilesional edema in left parietal and temporal lobes

**FIGURE 2:**
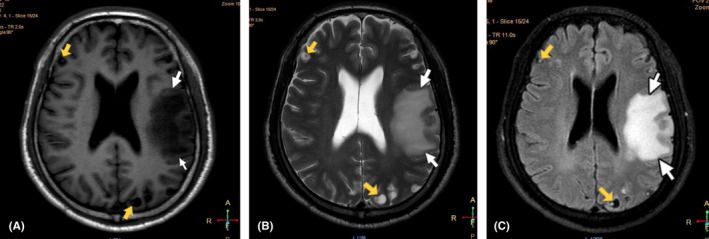
MRI brain. (A) Axial T1 weighted View. (B) Axial T2 weighted View. (C) Axial Flair View. White arrows showing marked amount of edema in left parietal and temporal lobes, Yellow arrows showing cystic lesions in bilateral cerebral and cerebellar hemispheres

The baseline investigations including complete blood examination, random blood sugar, renal function, and liver function tests were within reference range. The contrast‐enhanced computed tomography (CECT) scan of brain showed features suggestive of neurocysticercosis in varying stages with marked perilesional edema in left parietal and temporal lobe as shown in Figure 1. For the better visualization of edematous changes and cystic lesions, magnetic resonance imaging (MRI) of the brain (both plain and gadolinium enhanced) was performed. It revealed multiple NCC lesions on bilateral cerebrum and cerebellum with significant amount of edema in left parietal and temporal lobes as shown in Figure 2. There was no evidence of recent hemorrhage, infarction or mass in both CT and MRI scan. He was finally diagnosed as acute monoparesis secondary to multiple NCC.

**FIGURE 3:**
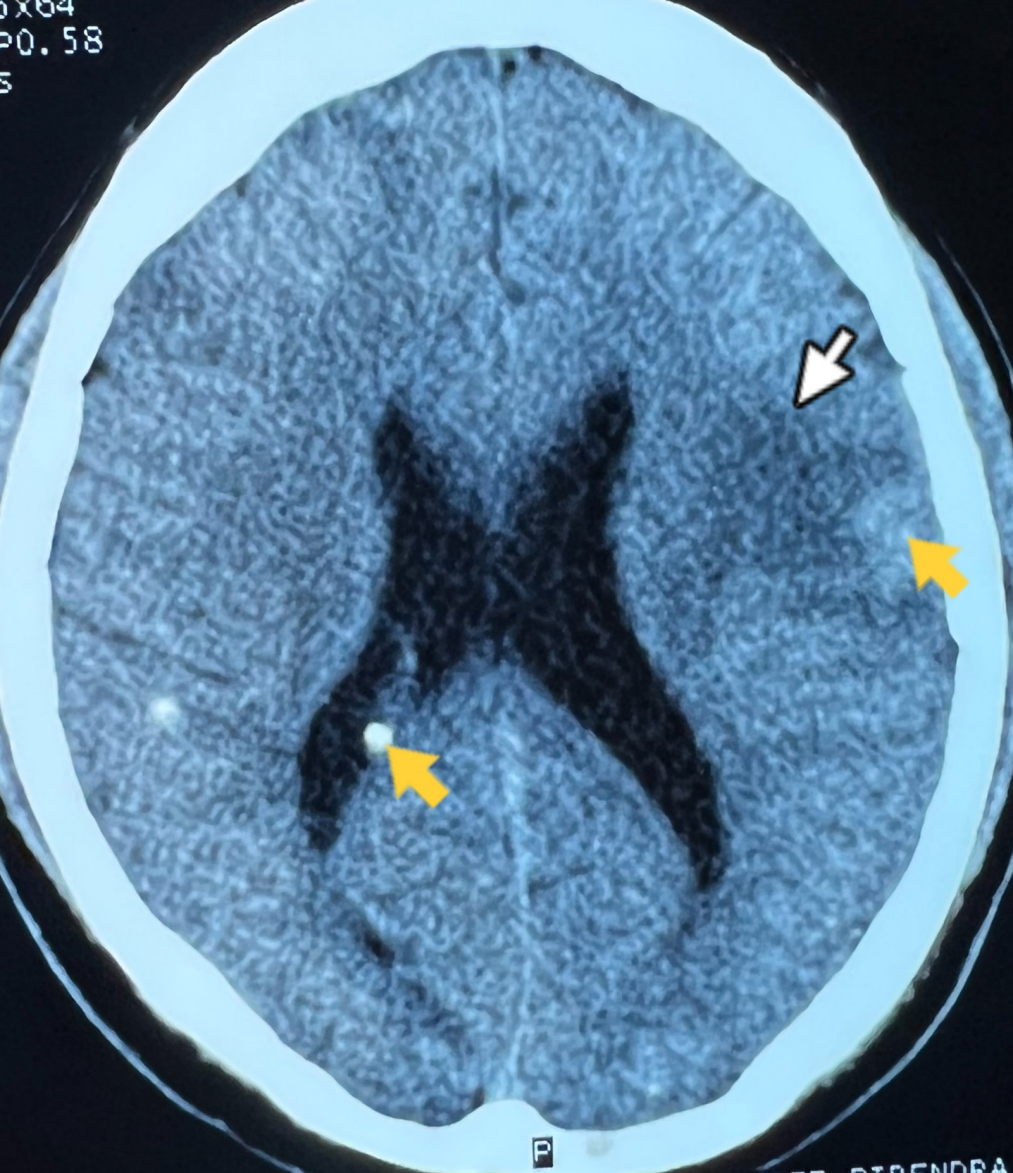
Repeat Axial Plain CT brain. White arrow showing significant reduction in edema as compared to the initial scan, Yellow arrow showing residual minute calcifications

He was then treated with tablet albendazole (400 mg twice a day) and tablet praziquantel (50 mg/kg/day in three divided doses) alongside steroid (intravenous dexamethasone 6 mg once a day) for 14 days. After the fifth day of treatment with albendazole and praziquantel, there was significant improvement in the clinical status of the patient. His motor examination on right upper limb was reverting back to normal with power of 5/5 on medical research council (MRC) grading and intact biceps and supinator reflexes. After the fourteenth day of treatment, repeat CT scan was done to evaluate the clinical improvement radiologically. There was significant reduction in edema as shown in Figure 3.

The final diagnosis at discharge was acute reversible monoparesis secondary to multiple NCC. He was then discharged on dexamethasone (2 mg three times a day) to be continued for 1 month with an advice for follow‐up after 1 month.

## 3 DISCUSSION

Neurocysticercosis (NCC) has been an endemic disease in many low‐ and middle‐income countries. According to World Health Organization (WHO) report, annually 2.5–8.3 million people get affected from NCC. And this has accounted for burden of 2.8 million disability‐adjusted life years (DALYs). The places where *Taenia* have been found endemic, 30% of epilepsy cases are ascribed to NCC.^6^ Seizures and headache are the two most common symptoms described in NCC. However, focal neurological deficits are also being reported in NCC in recent years.^1^, ^2^, ^3^, ^6^, ^7^ In between two variants of NCC, parenchymal NCC is the commonest to be found in most of the cases. However, extra‐parenchymal variant also tends to occur with intracranial hypertension, hydrocephalus, and arachnoiditis.^1^, ^2^, ^6^

The diagnosis of neurocysticercosis has various aspects including clinical examination, neuroimaging modalities and serological testing. Similarly, many diagnostic guidelines exist in the literature like those provided by Del Brutto et al.^8^ and Carpio et al..^9^ Above all, the gold standard modality for the diagnosis is biopsy or autopsy.^1^ The imaging modalities for NCC are computed tomography (CT) and magnetic resonance imaging (MRI) scans. The visibility of viable cystic lesions (scolex) and calcifications is typical of NCC. CT scan is the most common modality used to detect cysts and calcifications in NCC. However, for intraventricular and subarachnoid cysts MRI is used.^1^, ^6^, ^8^, ^9^ But the imaging modalities may not be available in all settings, especially in developing countries. As for our case, the typical cystic lesions at different stages were seen in multiple areas of brain in both CT and MRI scan.

Focal neurological deficits like pure motor hemiparesis (PMH) are caused by lacunar infarction. However, PMH has been reported in space occupying lesions throughout the recent years.^3^, ^4^, ^5^, ^10^, ^11^ The fact that space occupying lesions like NCC and mass in the brain can lead to ischemic cerebrovascular disease has been described extensively in the existing literature. The space occupying lesions affecting meninges lead to the thickening of leptomeninges. Due to this thickening, it produces inflammatory changes in adventitia of blood vessels forming circle of Willis. This will ultimately cause fibrosis and endothelial hyperplasia of blood vessels leading to occlusion, which is then followed by cerebral infarction.^3^, ^4^, ^10^, ^12^ With this process isolated manifestations like PMH can be manifested after the occlusion of perforating branches of major cerebral arteries. However, isolated monoparesis, as in our case, is a rare finding without history and evidence of ischemic cerebrovascular disease.

The majority of isolated pure monoparesis have been reported in the existing literature as a result of lacunar infarction.^11^, ^13^, ^14^, ^15^, ^16^, ^17^ However, according to a case series study, pure motor monoparesis is often caused by space occupying lesion rather than lacunar infarction.^5^ Monoparesis due to lacunar infarction have been found due to the involvement of superficial branches of cerebral arteries.^13^, ^15^, ^16^ Likewise, monoparesis described due to space occupying lesions also have been attributed to ischemic cerebrovascular disease as a result of the mechanism as described above. The space occupying lesions described are all mass lesions with surrounding edema, and none of the cases had reversible clinical course.^5^

In regard to our case, the brachial monoparesis did not account for any infarct etiology based on the neuroimaging findings. There was no evidence of recent infarction or hemorrhage in CT and MRI scan of brain. Multiple cystic lesions in bilateral cerebrum and cerebellum with marked perilesional edema, mostly in parietal and temporal region, were the findings in CT and MRI scan of brain. As there was no infarct or hemorrhagic evidence, the monoparesis in our case was probably due to the compressive effect on frontal and parietal lobe by moderate amount of perilesional edema. The local mass effect could be the reason behind the brachial monoparesis as reported in a case study.^10^

Majority of the cases, reported as monoparesis due to space occupying lesions, had irreversible clinical course. The final outcome was mortality in most of the cases.^5^ However, in our case, the patient had progressively improved clinical course during hospital stay after the therapy with praziquantel and albendazole. Our case was treated with praziquantel and albendazole alongside steroid based on the current consensus guidelines, where steroid was given to control edema and intracranial hypertension due to local inflammation from the death of larvae after the therapy.^18^The major limitation we had in our study was being unable to do a follow‐up with the patient after he was discharged from the hospital.

In conclusion, neurocysticercosis can present with wide variation of clinical symptoms and signs. As reported by us, reversible focal neurological deficit like monoparesis due NCC is an atypical phenomenon in the existing literature. Although space occupying lesions are seldomly reported with monoparesis, majority of them culminate in mortality. However, we report a case monoparesis in NCC with improving clinical and radiological outcome. This paves new way in understanding the etiology of focal neurological deficits other than infarcts and hemorrhages.

## AUTHOR CONTRIBUTIONS

BD contributed to case presentation, literature review, and writing the manuscript. SS contributed to literature review and editing the manuscript. Sheetal Shrestha had role in supervision, literature review, and editing manuscript. SA and AP edited the manuscript and contributed to data collection. AB and RP supervised the diagnosis and helped in building and editing the manuscript.

## FUNDING INFORMATION

The study did not receive any grant from funding agencies in the public, commercial, or not‐for‐profit sectors.

## CONFLICT OF INTEREST

The authors report no conflicts of interest.

## ETHICAL APPROVAL

This is a case report; therefore, it did not require ethical approval from ethics committee.

## CONSENT

Written informed consent was obtained from the patient for publication of this case report and accompanying images. A copy of the written consent is available for review by the editor‐in‐chief of this journal on request.

## REGISTRATION OF RESEARCH STUDIES

Not applicable.
